# Supplementary material for: Paternal low protein diet perturbs inter-generational metabolic homeostasis in a tissue-specific manner in mice
Source: Commun Biol. 2022 Sep 8;5:929. doi: 10.1038/s42003-022-03914-8 (PMC9458637; doi:10.1038/s42003-022-03914-8)
Supplement: Supplementary file 2 — Supplementary Information [file 42003_2022_3914_MOESM2_ESM.pdf]

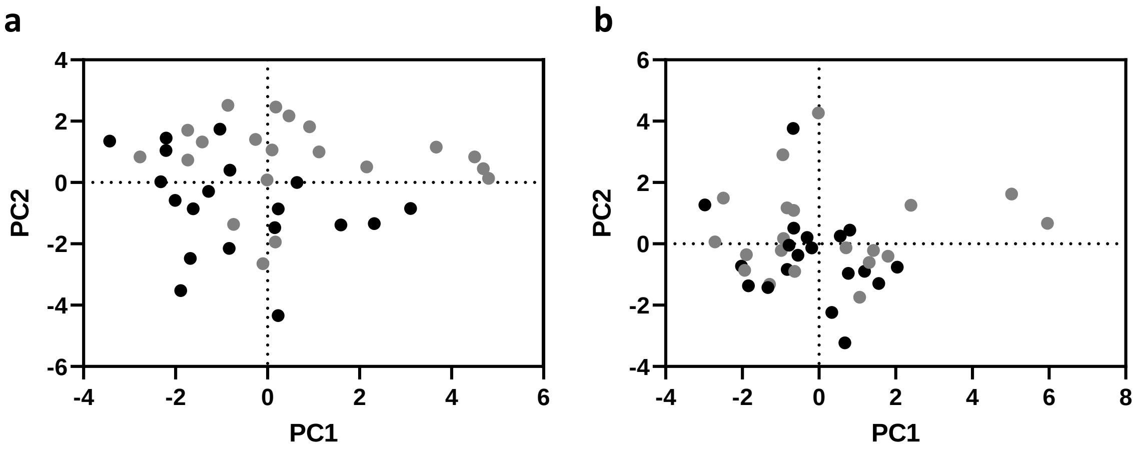

Supplemental Fig. 1: F1 neonatal tissue Principal Component Analysis: Principal component analysis (PCA) of differential lipids in NN, LL NL, LN and F1 neonatal offspring **a** liver and **b** serum. Grey circles are males and black circles are females. N = 9-10 offspring (4-5 males and 5 females) per treatment group.

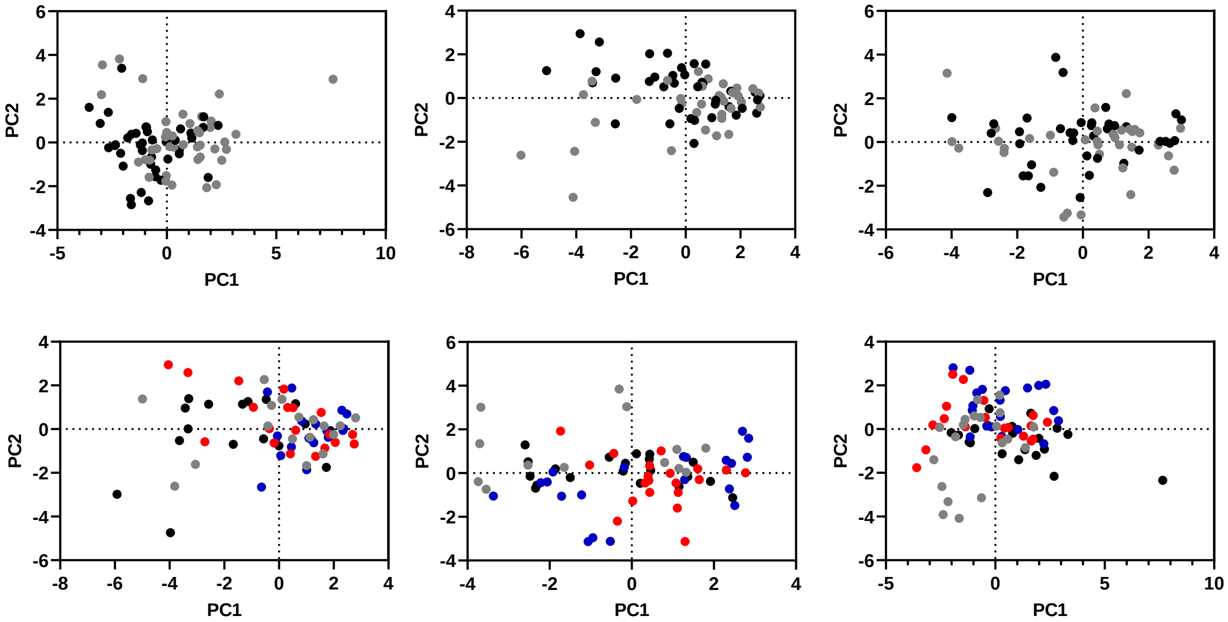

Supplemental Fig. 2; F1 adult tissue Principal Component Analysis: Principal component analysis (PCA) of differential lipids in F1 adult **a, d** liver, **b, e** gonadal fat and serum **c, f**. Grey circles in **a-c** are males and black circles are females. Black circles in **d-e** are NN offspring, grey circles are LL offspring, red circles are NL offspring and blue circles are LN offspring. N = 20 offspring (10 males and 10 females) sampled from all litters generated.

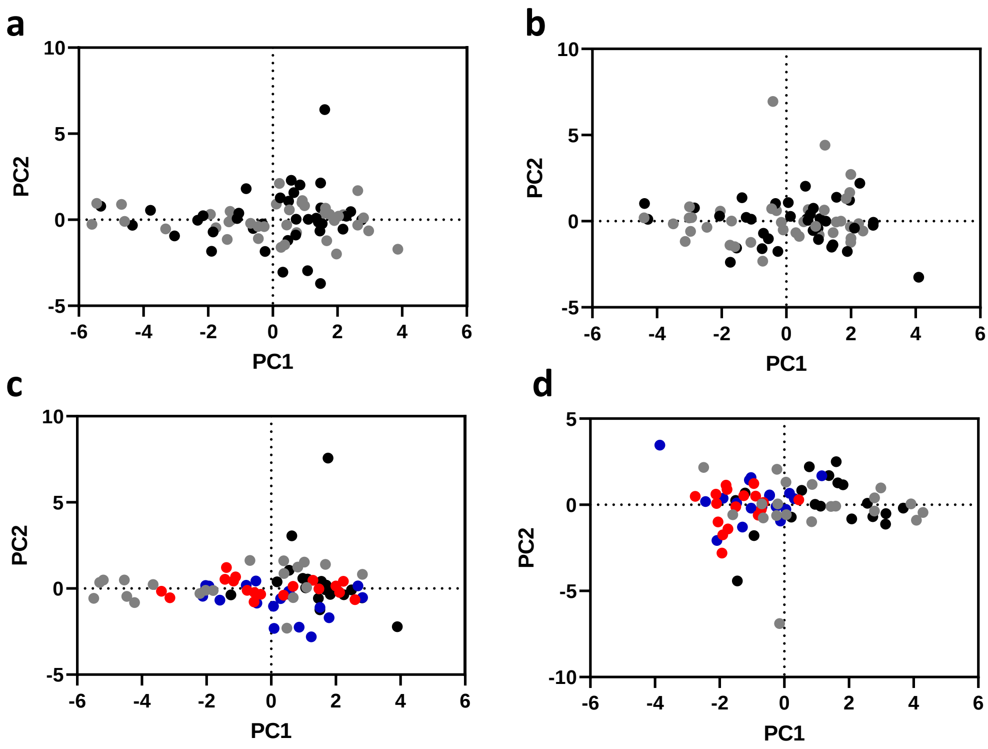

Supplemental Fig. 3; F2 neonatal tissue Principal Component Analysis: Principal component analysis (PCA) of differential lipids in F2 neonatal **a, c** liver and **b, d** serum. Grey circles in **a, b** are males and black circles are females. Black circles in **c, d** are NN offspring, grey circles are LL offspring, red circles are NL offspring and blue circles are LN offspring. N = 5-10 males and females per treatment group sampled from all litters generated.
