# Supplementary material for: Paternal low protein diet perturbs inter-generational metabolic homeostasis in a tissue-specific manner in mice
Source: Commun Biol. 2022 Sep 8;5:929. doi: 10.1038/s42003-022-03914-8 (PMC9458637; doi:10.1038/s42003-022-03914-8)
Supplement: Supplementary file 3 — Description of Additional Supplementary Files [file 42003_2022_3914_MOESM3_ESM.pdf]

Description of Additional Supplementary Files

File name: Supplementary Data 1
Description: F1 neonatal, adult and F2 neonatal body and organ weights and ELSIA/Assay tissue data

File name: Supplementary Data 2
Description: F1 neonatal offspring tissue lipidomic abundance

File name: Supplementary Data 3
Description: F1 adult offspring tissue lipidomic abundance

File name: Supplementary Data 4
Description: F1 adult offspring liver transcriptomic data

File name: Supplementary Data 5
Description: F1 adult male offspring liver transcriptomic data

File name: Supplementary Data 6
Description: F1 adult female offspring liver transcriptomic data

File name: Supplementary Data 7
Description: Adult liver Gene Ontology and Pathway output data

File name: Supplementary Data 8
Description: F1 adult fecal 16S sequencing data

File name: Supplementary Data 9
Description: F2 neonatal offspring tissue lipidomic abundance
