# Supplementary material for: Paternal low protein diet perturbs inter-generational metabolic homeostasis in a tissue-specific manner in mice
Source: Commun Biol. 2022 Sep 8;5:929. doi: 10.1038/s42003-022-03914-8 (PMC9458637; doi:10.1038/s42003-022-03914-8)
Supplement: Supplementary file 13 — Reporting Summary [file 42003_2022_3914_MOESM13_ESM.pdf]

nature portfolio

Corresponding author(s): COMMSBIO-22-0854A

Last updated by author(s): Aug 18, 2022

Reporting Summary

Nature Portfolio wishes to improve the reproducibility of the work that we publish. This form provides structure for consistency and transparency in reporting. For further information on Nature Portfolio policies, see our Editorial Policies and the Editorial Policy Checklist.

Statistics

For all statistical analyses, confirm that the following items are present in the figure legend, table legend, main text, or Methods section.

n/a	Confirmed	
☐	☒	The exact sample size (n) for each experimental group/condition, given as a discrete number and unit of measurement
☐	☒	A statement on whether measurements were taken from distinct samples or whether the same sample was measured repeatedly
☐	☒	The statistical test(s) used AND whether they are one- or two-sided<br>*Only common tests should be described solely by name; describe more complex techniques in the Methods section.*
☐	☒	A description of all covariates tested
☐	☒	A description of any assumptions or corrections, such as tests of normality and adjustment for multiple comparisons
☐	☒	A full description of the statistical parameters including central tendency (e.g. means) or other basic estimates (e.g. regression coefficient) AND variation (e.g. standard deviation) or associated estimates of uncertainty (e.g. confidence intervals)
☒	☐	For null hypothesis testing, the test statistic (e.g. F, t, r) with confidence intervals, effect sizes, degrees of freedom and P value noted<br>*Give P values as exact values whenever suitable.*
☒	☐	For Bayesian analysis, information on the choice of priors and Markov chain Monte Carlo settings
☐	☒	For hierarchical and complex designs, identification of the appropriate level for tests and full reporting of outcomes
☐	☒	Estimates of effect sizes (e.g. Cohen's d, Pearson's r), indicating how they were calculated

Our web collection on statistics for biologists contains articles on many of the points above.

Software and code

Policy information about availability of computer code

Data collection	Raw high-resolution mass-spectrometry data were processed using XCMS (www.bioconductor.org) and Peakpicker v 2.0 (an in-house R script). 31P NMR data was processed using TopSpin 4.0.7. OriginLab 2018, Excel 2016, GraphPad PRISM 9 and SPSS 24 were used for data curation, statistical analysis and preparation of figures.
Data analysis	N/A

For manuscripts utilizing custom algorithms or software that are central to the research but not yet described in published literature, software must be made available to editors and reviewers. We strongly encourage code deposition in a community repository (e.g. GitHub). See the Nature Portfolio guidelines for submitting code & software for further information.

Data

Policy information about availability of data

All manuscripts must include a data availability statement. This statement should provide the following information, where applicable:

- Accession codes, unique identifiers, or web links for publicly available datasets
- A description of any restrictions on data availability
- For clinical datasets or third party data, please ensure that the statement adheres to our policy

All data in the present study are provided as Electronic Supplementary Material.

Human research participants

Policy information about studies involving human research participants and Sex and Gender in Research.

Reporting on sex and gender	N/A
Population characteristics	N/A
Recruitment	N/A
Ethics oversight	N/A

Note that full information on the approval of the study protocol must also be provided in the manuscript.

Field-specific reporting

Please select the one below that is the best fit for your research. If you are not sure, read the appropriate sections before making your selection.

☒ Life sciences ☐ Behavioural & social sciences ☐ Ecological, evolutionary & environmental sciences

For a reference copy of the document with all sections, see nature.com/documents/nr-reporting-summary-flat.pdf

Life sciences study design

All studies must disclose on these points even when the disclosure is negative.

Sample size	Samples sizes were determined using power calculations using previously published data from our group.
Data exclusions	No data were excluded from the study.
Replication	The number of sires used was based on prior power calculations using our previously published data to ensure replication of model phenotypic effects. Tissues were sampled from across several litters (and therefore several fathers) which all showed similar phenotypic changes in growth and metabolic status. For the analysis of tissue lipid profiles, all the tissue was used during the analytical procedures and so no technical replicates could be performed. Finally, these data are in line with our previous published studies using this model giving confidence in the validity of the data.
Randomization	Mice were randomly assigned to their dietary groups after acclimatisation to the facility. Mass spectrometry samples were run in random order.
Blinding	Sample names afforded partial blinding, though as around 1100 mass spectrometry samples were run in a high throughput manner in a random order, further blinding was not considered necessary.

Reporting for specific materials, systems and methods

We require information from authors about some types of materials, experimental systems and methods used in many studies. Here, indicate whether each material, system or method listed is relevant to your study. If you are not sure if a list item applies to your research, read the appropriate section before selecting a response.

Materials & experimental systems

n/a	Involved in the study	
☒	☐	Antibodies
☒	☐	Eukaryotic cell lines
☒	☐	Palaeontology and archaeology
☐	☒	Animals and other organisms
☒	☐	Clinical data
☒	☐	Dual use research of concern

Methods

n/a	Involved in the study	
☒	☐	ChIP-seq
☒	☐	Flow cytometry
☒	☐	MRI-based neuroimaging

Animals and other research organisms

Policy information about studies involving animals; ARRIVE guidelines recommended for reporting animal research, and Sex and Gender in Research

Laboratory animals	C57BL/6 mice
Wild animals	The study did not involve wild animals
Reporting on sex	Both sexes were analyzed in our study and the impact of sex was incorporated into our statistical analysis. Where sex was found to have a significant influence on the variable reported the data for both sexes were presented separately.
Field-collected samples	The study did not involve samples collected from the field.
Ethics oversight	All procedures were conducted in accordance with the UK Home Office Animal (Scientific Procedures) Act 1986 and local ethics committees at Aston University.

Note that full information on the approval of the study protocol must also be provided in the manuscript.
